# Hypertension and Metabolic Syndrome in Persons with HIV

**DOI:** 10.1007/s11906-020-01089-3

**Published:** 2020-09-03

**Authors:** Sepiso K. Masenga, Fernando Elijovich, John R. Koethe, Benson M. Hamooya, Douglas C. Heimburger, Sody M. Munsaka, Cheryl L. Laffer, Annet Kirabo

**Affiliations:** 1 HAND Research group, School of Medicine and Health Sciences, Mulungushi University, Livingstone, Zambia; 2 Department of Biomedical Sciences, School of Health Sciences, University of Zambia, Lusaka, Zambia; 3 Department of Medicine, Vanderbilt University Medical Center, Nashville, TN USA; 4 School of Public Health, University of Zambia, Lusaka, Zambia; 5 Department of Internal Medicine, School of Medicine, University of Zambia, Lusaka, Zambia; 6 Department of Molecular Physiology and Biophysics, Vanderbilt University School of Medicine, Room 536 Robinson Research Building, Nashville, TN 37232-6602 USA

**Keywords:** HIV, Metabolic syndrome, Hypertension, Antiretroviral therapy, Immune modulators

## Abstract

**Purpose of Review:**

With the advent of highly active antiretroviral therapy (ART), the life span of persons with HIV (PWH) has been nearly normalized. With aging, prevalence of the metabolic syndrome (MetS), including hypertension, has increased in the HIV population and exceeds that in the general population in some studies. This is due to a combination of traditional risk factors in addition to the effects attributable to the virus and ART. We review recent findings on the mechanisms contributing to MetS and hypertension in PWH, particularly those specific to the viral infection and to ART.

**Recent Findings:**

Activation of the renin-angiotensin-aldosterone system (RAAS) and chronic immune activation contribute to the development of MetS and hypertension in PWH. HIV proteins and some ART agents alter adipocyte health contributing to dyslipidemias, weight gain, and insulin resistance. HIV infection also contributes to hypertension by direct effects on the RAAS that intertwine with inflammation by the RAAS also contributing to T cell activation.

**Summary:**

Recent data suggest that in addition to current ART, therapeutic targeting of the MetS and hypertension in PWH, by interfering with the RAAS, treating insulin resistance directly or by use of immunomodulators that dampen inflammation, may be critical for preventing or treating these risk factors and to improve overall cardiovascular complications in the HIV-infected aging population.

## Introduction

HIV affects about 1.2 million persons in the USA [1]. The introduction of antiretroviral therapy (ART) has tremendously improved survival among persons with HIV (PWH), providing a nearly normal life span. However, aging PWH have a heightened risk for metabolic disorders, including diabetes mellitus and dyslipidemia. Because knowledge on the pathogenesis of metabolic disorders was acquired in HIV-negative populations, it is not clear whether the same etiology and pathogenesis operate in PWH. The metabolic complications of PWH were initially attributed to long-term antiretroviral therapy (ART), but more recent evidence suggests that the viral infection itself could play a role, both leading to heightened risk for metabolic syndrome (MetS) in PWH [2••,^3^••].

The prevalence of MetS in PWH ranges from 11 to 48% and is particularly high in sub-Saharan Africa [4–6,7••,^8^•,^9^•,^10^,^11^••,^12^,^13^,^14^•]. Some studies report higher prevalence in females [5, 6, 13, 15] and others in males [4]. Regardless of prevalence, the risk of CVD seems greater in HIV+ women than in HIV+ men [15]. This may be due to higher prevalence of obesity and MetS in women, to suboptimal management of their dyslipidemia and hypertension [16], or to effect of sex hormones or differential fat distribution between sexes [17••]. Men accrue more adipose tissue in the subcutaneous layers of the trunk and abdomen (android fat), whereas women accumulate more visceral adipose tissue (gynoid fat accumulation around the hips and buttocks), particularly when postmenopausal as pre-menopause women have less visceral adipose tissue compared with men [18, 19]. Therefore, the waist circumference cutoffs used in the definition of MetS may underestimate risk of CVD in women, and most importantly, it is well known that abdominal visceral adipose tissue is a better predictor for CVD and metabolic derangements compared with subcutaneous adipose tissue.

MetS is a cluster of disorders characterized by high waist circumference (> 102 cm for men; > 88 cm for females), high triglycerides (≥ 150 mg/dL or ≥ 1.69 mmol/L), low high-density lipoprotein cholesterol (HDL-c) (men < 40 mg/dL or < 1.0 mmol/L; women < 50 mg/dL or < 1.3 mmol/L), high blood pressure (≥ 130/≥ 85 mmHg), and high fasting glucose (≥ 110 mg/dL or ≥ 6.1 mmol/L) and is a major risk factor for CVD and type 2 diabetes [20].

The prevalence of hypertension is also increased in PWH treated with ART. The interactions between HIV, hypertension, and MetS are not clearly understood. We will discuss recent findings about the gender differential mechanisms contributing to hypertension and MetS in PWH.

## Metabolic Syndrome in People Living with HIV versus the General Population

Although it is clear that HIV infection and ART affect all components of MetS, it is still controversial whether the incidence and prevalence of MetS are higher in PWH than in the general population [8•,^9^•,^11^••], possibly reflecting methodological or demographic differences among reports. A recent meta-analysis [11••] showed that the burden of MetS is rapidly growing in both PWH and the general population, suggesting that other risk factors such as diet, lifestyle, age, genetic predisposition, and the presence of other comorbidities are important in both populations. A Polish study recently showed that in PWH, age ≥ 40 and nadir CD4 counts (< 350 cells/mm3) were HIV-specific risk factors for MetS [4].

## Role of HIV Infection and ART in Metabolic Syndrome

As stated above, both HIV infection and effects of ART were shown to be independent risk factors for MetS in PWH by some [2••] but not all investigators [6]. The controversy may stem from the fact that ART is started immediately after the diagnosis of HIV infection in most countries, with a consequent dearth of literature on the role of HIV infection as an isolated risk factor for MetS.

Effects of HIV infection and ART treatment on adipose maturation, cytokine signaling, and expression of adipocyte regulatory genes such as PPAR-γ [21••] alter adipose tissue morphology, distribution, and metabolism, resulting in the lipodystrophy phenotype in PWH [3••]. This is characterized by central lipohypertrophy (increased visceral fat deposition) and lipoatrophy (loss of limb fat), the reason by which waist circumference is a better marker for MetS in PWH since body mass index does not account for the fat distribution abnormality [21••]. The consequences of lipodystrophy are not fully understood but may include a contribution to the inflammatory state of PWH, perhaps via adipocyte-specific defects that modify the phenotype of immune cells. The accumulation of visceral adipose tissue is one of the main drivers of increased inflammation related to body composition. Adipocytes constitutively express IL-6 and tumor necrosis factor-alpha, which then drain to the liver via the portal vein and stimulate Kupffer cells to produce C-reactive protein and other inflammatory markers.

A role has been described for the HIV accessory protein viral protein R (Vpr), in dysregulating metabolically critical transcription factors that cause abnormal adipose differentiation, turnover, and function. Mice that produced Vpr in tissues that sequester HIV after ART inhibited PPARγ target gene expression and activated glucocorticoid target gene expression [22], with development of adipocyte dysfunction. Interestingly, these mice recapitulated typical features of the human syndrome including accelerated lipolysis and increased macrophage infiltration of adipose tissue, suggesting that chronic viral infection may be a causative factor in the pathogenesis of HIV lipodystrophy.

In terms of effects of ART on the metabolic profile of PWH, there has been some controversy regarding differential effects of different drugs. However, it is well established that the protease inhibitors (PIs), particularly lopinavir/ritonavir and indinavir but less so some newer agents, exert profound effects on triacylglycerols and lipid transport. This leads to dyslipidemias, lipodystrophy, and insulin resistance and promotes adipokine secretion, with consequent vascular inflammation and endothelial dysfunction.

Among the nucleoside reverse transcriptase inhibitors (NRTIs), the use of tenofovir disoproxil fumarate (TDF) and abacavir is associated with lower risk for developing MetS compared with zidovudine although extended use of TDF combined with PIs may be nephrotoxic [3••].

Combination therapy with NRTIs and non-nucleoside reverse transcriptase inhibitors (NNRTIs) was associated with more severe stiffening of large elastic arteries in subjects with MetS than that in those without MetS or in those not receiving combined therapy [10], suggesting that regardless of the combination’s contribution to MetS, it definitely exaggerates arterial remodeling.

Insulin resistance with impaired glucose metabolism has been observed with PIs (indinavir and lopinavir/ritonavir), NRTIs (stavudine, zidovudine, lamivudine, and didanosine), and NNRTIs (efavirenz) [14•]; its underlying mechanisms are not fully understood, but in the case of indinavir, it involves blockade of the insulin-sensitive glucose transporter GLUT4 [14•]. Recently, more weight gain on integrase strand transfer inhibitors (INSTIs) compared with NNRTI-based regimens has been reported [23]. In this class of INSTIs, dolutegravir and raltegravir induced adipogenesis, lipogenesis, oxidative stress, fibrosis, and insulin resistance in PWH [24].

HIV infection and ART-induced MetS may involve different mechanisms as supported by the fact that in many studies MetS is more common in PWH on ART than in untreated subjects [25]. In a large cross-sectional study in Kenya, although the prevalence of MetS was not different between ART-treated and naïve PWH [9•], and although the major risk factors for MetS were those in non-HIV populations (age, female sex, and BMI), there were nonetheless phenotypic differences between groups, such as more hyperglycemia and reduced HDL cholesterol in those on ART. Finally, and most importantly, it has been reported that 37% of PWH with MetS at onset of ART no longer met the criteria for this diagnosis 2 years after therapy [26]. This was largely due to increases in HDL cholesterol, without concomitant improvement in hypertension over 96 weeks. Obesity at start of ART was an independent predictor of lack of MetS regression.

A mechanism that may be specific for ART-induced MetS is drug-induced oxidative DNA damage [7••]. This was assessed by measurement of plasma levels of 8-hydroxy-2-deoxyguanosine (8-OHdG) in ART-treated and ART-naïve PWH. Levels were increased in the former and conferred an increased odd ratio of ~23 for MetS, particularly on subjects treated with PIs. Therefore, the increased life span of PWH by ART may be associated with detrimental effects of DNA damage on CVD risk, mediated by endothelial, immune, and adipose cell dysfunction with consequent atherosclerosis.

## Role of Inflammation

Adipose tissue is a reservoir for HIV and contains unique activated immune cells. Replication-competent HIV has been isolated from adipose-derived CD4+ T cells and adipose tissue stromal vascular cells [27–29]. Within adipose tissue, immune cells including T cells and macrophages are more susceptible to HIV and release viral proteins at high concentrations, which then affect adipocytes [30, 31]. However, it is not known how or where immune cells are activated in HIV-associated MetS. Key unknowns include the distribution of these activated immune cells in different fat deposits and whether adipose resident antigen-presenting cells (APCs) such as macrophages and dendritic cells drive increased adipose tissue inflammation compared with their effects in HIV-negative persons. Also unknown is the antigenic site for immune cell activation, the extent to which APCs are recruited or proliferate within target adipose tissue depots during HIV infection and whether these cells can exit sites of activation and localize to other tissues.

Another important factor altering adipose tissue health with consequential effects on the vasculature is the recruitment of activated monocytes to adipose tissue, where they differentiate into macrophages. This chemotaxis may be adaptive, for phagocytosis of necrotic or necrotizing adipocytes, the consequence of their rapid expansion with consequent hypoxic death [32]. Once in the adipose tissue, macrophages produce pro-inflammatory cytokines, including interleukin (IL)-6, IL-1β, and tumor necrosis factor-alpha (TNF-∝) that alter adipose function and induce endothelial dysfunction even after total HIV viral suppression [32]. In addition, there is also the issue of variable penetration of ART particularly the NRTIs such as TDF, emtricitabine, abacavir, and lamivudine into adipose tissue, which could contribute to low-level viral replication in the tissue [33].

## Use of Immunomodulators in HIV

A persistent state of inflammation and chronic immune activation is the hallmark for metabolic derangements that result in hypertension and MetS [3••]. It has been argued that chronic inflammation may underlie the higher risks for MetS, diabetes, CVD, and cancer of ART-treated PWH compared with ART-naïve subjects observed in some studies [3••]. Several drug therapies with immune-modulatory effects have been shown to reduce inflammation and the risk for metabolic derangements in PWH when used in conjunction with ART. For example, the purinergic P2X receptor inhibitors NF449 and A438079 had the dual effects of blocking HIV-1 infection and reducing HIV-1-stimulated IL-10 and IL-1 production in human tonsil cells [3••]. Statins have also been shown to reduce inflammatory markers by inhibiting immune activation and expression of co-stimulatory molecules on antigen-presenting cells and T cells [3••]. Other drugs that reduce inflammation by different mechanisms, such as canakinumab, sevelamer carbonate, chloroquine, and hydroxychloroquine [3••], have been shown to reduce inflammation and the risk for MetS and CVD in HIV.

## Role of Hypertension

In many studies, hypertension is more prevalent in ART-treated than in ART-naïve PWH and in ART-naïve than in HIV-negative subjects [34•]. In the HIV-HY study, prevalence of established plus pre-hypertension was 41.6%, with only two-thirds aware and one-third controlled despite the use of mono or combined therapies, with three-quarter of patients receiving antagonists of the RAS [35]. In PWH with MetS, prevalence of hypertension may reach 96%, and once established, it is an important risk factor for acute myocardial infarction [2••]. Aging, the metabolic abnormalities, endothelial dysfunction, and inflammation of HIV infection and ART treatment for longer than 2 years are the drivers of hypertension in PWH [2••]. Some have shown that central obesity and overweight increased the risk of hypertension and MetS in PWH as they do in the general population [9•], whereas others reported that nadir CD4 T cell counts were also associated with hypertension in European [35] and African [34•] PWH, a finding specific to HIV populations. In addition to HIV-related inflammation and detrimental effects of ART on adipocyte health and lipid metabolism, specific components of the virus such as the negative factor (Nef) protein, transcription protein (Tat), and glycoprotein 120 may contribute to the link between MetS and hypertension in PWH, perhaps superimposed on individual genetic predisposition [36–38].

There is growing evidence that hypertension is an immune-related condition with various non-immune interplaying factors. We have shown that in PWH, hypertension is associated with increased levels of inflammatory (IL-6, IL-17, tumor necrosis factor-alpha receptor 1, IL-5, intercellular adhesion molecule 1, macrophage inflammatory protein-1 alpha, elevated eosinophils) and with immune-activation markers independently from the other components of MetS [34•,^39^••]. Inflammatory markers such as hsCRP and IL-6 are increased in PWH with MetS compared with those without MetS [2••], suggesting that inflammation of MetS plays a role in the pathogenesis of hypertension in PWH.

Because dyslipidemia and hypertension are major features of MetS and also the core drivers of CVD risk, their prevention is of the utmost importance for PWH [25]. This requires monitoring of the underlying factors associated with hypertension, such as immune status, inflammation, dietary salt intake, and metabolic abnormalities, especially with longer duration on ART. Regarding the latter, there seem to be differences in the detrimental effects of some agents on metabolic versus blood pressure abnormalities. For example, PI-containing regimens were associated with worse dyslipidemia but with less elevation of diastolic blood pressure in a Ugandan series [40].

## The Renin-Angiotensin System

The renin-angiotensin-aldosterone system (RAAS) is a major regulator of blood pressure and fluid balance. The renin-ACE-Ang II-AT1 receptor pathway is responsible for the major pressor physiological actions of vasoconstriction, Na + retention, and aldosterone release, which become detrimental in cardiovascular diseases such as hypertension and heart failure. In contrast, the renin-ACE-Ang II-AT2 and the renin-ACE2/neprilysin-Ang 1–7-mas receptor pathways exert opposite effects that modulate the overall effect of RAAS in normal physiology and may be beneficial in disease states. An analogous balance between the two axes occurs in the tissue RAAS. In the kidney, the local RAAS is activated by reduced renal function and involved in the progression of renal injury via AT1 receptor activation. This produces glomerular hyperfiltration, interstitial and vascular inflammation, cell growth, migration, differentiation, and apoptosis and activation of signaling pathways that lead to tissue damage. In humans, large studies of ACEIs and ARBs reduced proteinuria and slowed the progression of diabetic and nondiabetic nephropathies beyond what is expected from their BP lowering. Aldosterone and activation of the mineralocorticoid (MCR) and GPR30 receptors also contribute to renal tubule and glomerular oxidative stress, inflammation, and fibrosis, which can be improved with MCR blockers.

Interactions between the RAAS and HIV infection have been described at several levels. For example, renin is known to enhance HIV replication within T cells via activation of NFkappaB and PI3K pathways [41••]. Also, drugs that are used because they block renin (e.g., aliskiren) or the HIV protease (e.g., duranavir) also block the alternate enzyme, which may be relevant for the development of agents with dual action that address both the HIV infection and its cardiovascular and metabolic complications [42].

In HIV-infected subjects, serum levels of ACE are elevated, compared with controls [43] and physiological activation of the RAAS by dietary salt-restriction is associated with activation of immune markers that does not occur in HIV-negative subjects [44]. RAAS activation is a major driver of tissue fibrosis, which impairs CD4+ T cell recovery in lymph nodes and contributes to metabolic abnormalities by affecting adipose tissue in HIV-infected subjects. However, telmisartan was unable to improve fibrosis of these tissues beyond the benefit observed during ART therapy [45].

In terms of renal disease, HIV induces podocyte injury via downregulation of the vitamin D receptor (VDR), which is attributable to epigenetic effects of the virus on the VDR promoter [46••]. This is enhanced if there is concomitant hyperglycemia [47] and results in activation of the renal RAAS. The latter leads to an increase in reactive oxygen species and tubular cell DNA injury that can be attenuated by vitamin D, vitamin D agonists, tempol, and losartan [47, 48]. Mice with HIV nephropathy and selective knockout of the AT1 receptor in podocytes still derive benefit from AT1 blockers, suggesting that these agents may also benefit vascular abnormalities of HIV nephropathy [49]. Finally, another pathway that contributes to the development of HIV nephropathy is RAAS-dependent phosphorylation and activation of mTOR. Interestingly, this is blocked by AT2 but not AT1 antagonists, demonstrating the involvement of the putatively “protective” AT2 receptor in the pathogenesis of HIV nephropathy [50]. In view of all the findings above, it is not surprising that ACE inhibitors [51] and AT1 receptor blockers [52] are protective of progressive proteinuria and renal dysfunction in subjects with HIV nephropathy.

Although much has been learned about the participation of the RAAS in the pathogenesis of MetS in the population at large [53], not much is known about these interactions in the HIV-infected population. Plasma levels of aldosterone, which are somewhat increased during salt depletion in PWH compared with control subjects, predict insulin resistance in multivariate analyses controlled for adiponectin and visceral adipose tissue [54]. Moreover, levels of natriuretic peptides, which are physiological downregulators of aldosterone action, are decreased in HIV-infected patients, compared with controls. They correlate inversely with aldosterone levels, clinical parameters of MetS, and the HOMA index of insulin sensitivity, indicating that a deficit in these peptides plays a role in determining aldosterone hyperactivity with its detrimental metabolic consequences in HIV subjects [55]. Additional stimulation of the mineralocorticoid receptor in HIV subjects with lipodystrophy may be due to cortisol, leading to a lipid profile similar to that in Cushing’s syndrome. This is due to the fact that lipodystrophic subcutaneous fat overexpresses 11-beta-hydroxy-dehydrogenase, with augmented regeneration of cortisol by adipocytes and increased urinary cortisol/cortisone ratios. Levels of 11-beta-hydroxy-dehydrogenase mRNA correlate with insulin resistance in these subjects [56]. The observations above would suggest that mineralocorticoid receptor blockade could be beneficial for HIV-induced Mets. However, a study of eplerenone showed improvement in some inflammatory markers but not in insulin resistance in HIV subjects with MetS [57].

**Fig. 1:**
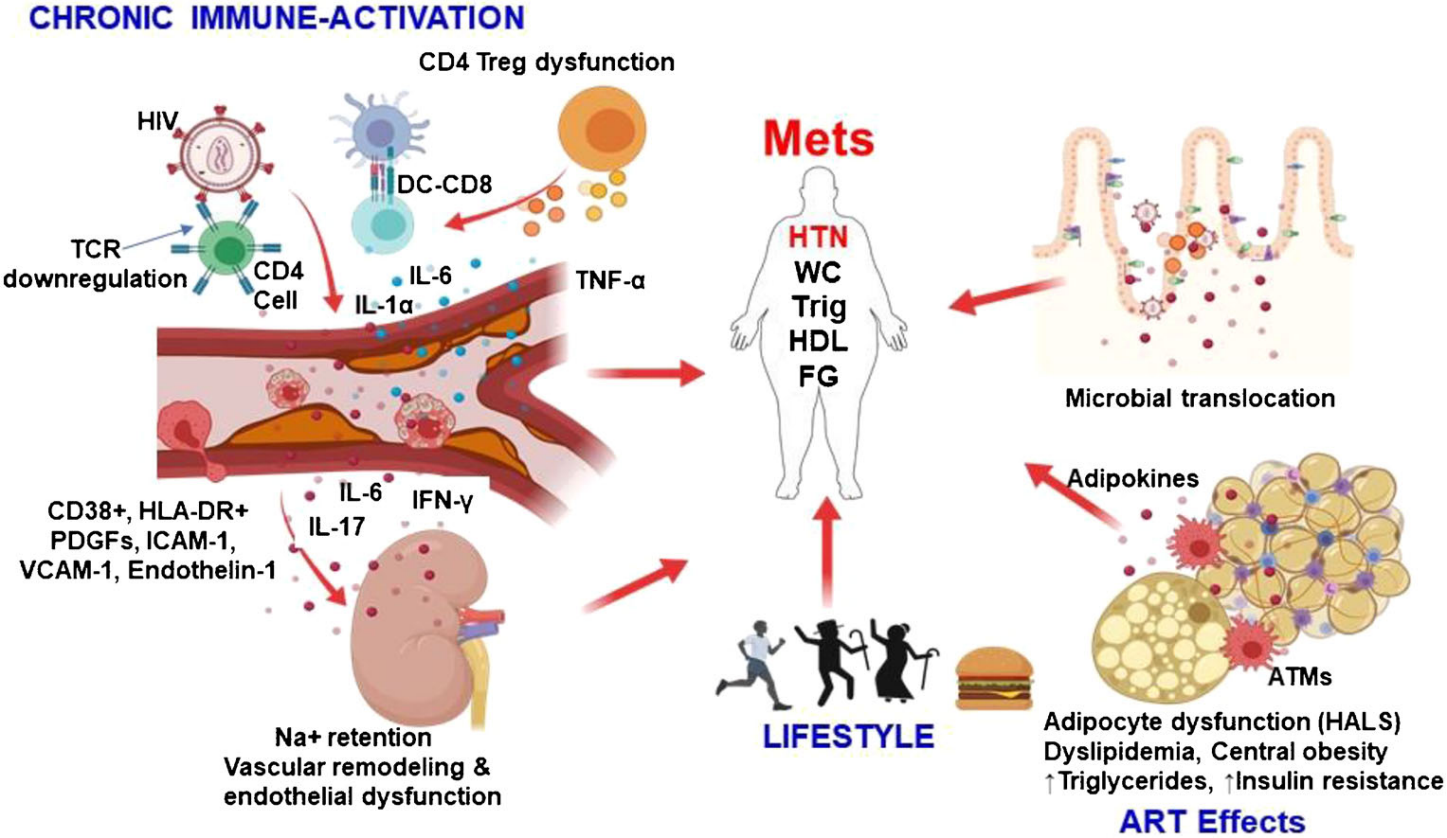
HIV-related chronic immune activation, ART effects, and lifestyle contribute to hypertension and metabolic syndrome. Infected dendritic cells (DCs) through toll-like receptor (TLR) stimulation activate CD8 cytotoxic cells that have direct vascular effects on the endothelial cells. Immune-activation is accompanied by expression of CD38+ and human leukocyte antigen D-related (HLA-DR) expression on T cells, markers associated with disease progression. Activated endothelial cells increase the expression of platelet derived growth factors, intercellular adhesion molecule-1 and vascular adhesion molecule-1 which increases leukocyte adhesion to the vessel producing a fertile environment for atherosclerotic events. Endothelin-1 produced by endothelial cells leads to vasoconstriction. Binding of HIV to CD4 cells through the glycoprotein (gp) 120/41 downregulates T cell receptor (TCR) leading to cell death. CD4 T-regulatory (Treg) cells responsible for induction of anti-inflammatory IL-10 is rendered dysfunctional in HIV leading to overactivation of immune system resulting in persistent chronic immune activation. Microbial translocation into blood circulation due to depleted CD4 cells in the gut further exacerbate the inflammation. All these vascular events contribute to hypertension through direct damage from cells and inflammatory cytokines such as IL-17 and IL-6. T cells infiltrate the kidneys producing inflammatory cytokines resulting in increased sodium retention, vascular remodeling, and endothelial dysfunction and hypertension. Effects of ART causes HIV-associated lipodystrophy syndrome (HALS). Increased adipose tissue macrophage infiltrate the adipocytes resulting in secretion of adipokines that induce increased insulin resistance. In addition, lifestyle factors such as aging and diet synergize with ART effects and chronic immune activation and contribute to MetS components especially hypertension and increase risk of MetS

Summary of the immune mechanisms operating in MetS and hypertension of PWH Fig. 1 shows the excessive and prolonged stimulation of cells of both the innate and adaptive immune system, which provoke chronic inflammation that persists even after HIV viral suppression. We have shown that this may damage other cells, tissues, and the kidney vasculature in direct manner and also indirectly, through cytokine production that contributes to atherosclerosis and hypertension. These vascular effects, coupled with HIV-associated lipodystrophy (HALS), characterized by predominant central obesity and with ART-induced metabolic abnormalities, including hypertriglyceridemia, decreased HDL-C, reduced insulin sensitivity, and higher plasma glucose, combined with and synergize traditional lifestyle risk factors (e.g., aging and obesity) to determine high prevalence of MetS and hypertension in PWH.

## Conclusion

Novel discoveries have broadened our understanding of the roles played by HIV infection and ART on development of MetS and hypertension in PWH. However, there is a paucity of studies in this field and much remains to be investigated to unravel the mechanisms by which the multiple components of MetS develop. Despite the lack of specific therapeutic guidelines applying this new knowledge, it has become clear that close monitoring of patients on ART for each component of MetS is critical to attempt prevention or treatment as they develop. In this regard, the use of immunomodulators concomitantly with ART may be a potential therapeutic approach to mitigate development of MetS, hypertension, and their cardiovascular complications in PWH.
